# On the Changes Induced in the Situation and Structure of the Internal Organs, under Varying Circumstances of Health and Disease; and on the Nature and External Indications of These Changes

**Published:** 1845-01

**Authors:** 


### ART. XI.

*On the Changes induced in the Situation and Structure of the Internal Organs, under varying circumstances of Health and Disease; and on the Nature and external Indications of these Changes.* By FRANCIS SIBSON. (*Transactions of the Provincial Medical and Surgical Association*, Vol. XII, pp. 307-574.)—*London*, 1844.

MR. SIBSON commences by informing his readers that he some years past discovered that his notions of the "normal and healthy sites of the various viscera were ill defined." Few persons can have intently pursued the investigation of thoracic disease without feeling, to a greater or less amount, the pressure of the difficulty under which this young inquirer suffered; and fewer still would have conceived, or above all steadily carried out for a lengthened period, the plan he employed to remove this difficulty in future investigations conducted either by himself or others. This plan and its results are described in the paper which we propose to analyse—a paper no less remarkable for its originality and modesty of style than the solid information it conveys. We set a very high value on this production, and we anticipate great things from the future labours of its author. We intend paying him a compliment in subjecting his treatise to a close critical examination, and we trust he will receive our criticisms in the spirit in which they are offered.

Mr. Sibson set out by taking diagrams of the position of the viscera, as discovered on post-mortem examination; first, drawing an outline of the ribs and sternum, aud then adding the internal viscera, taking care that their bearings to each other and the ribs were accurately planned. After a time he procured a frame and stretched strings across and along it, at distances from each other of three inches, (the whole frame was thus subdivided into forty-five squares;) and ruled a piece of paper with squares of a like fashion, but of one third the size. When the outlines of the viscera of any subject were to be traced, the frame was laid over the body and the objects behind each three-inch square accurately marked on the corresponding one inch square on the paper.

Subsequently, a further modification was adopted at the suggestion of Dr. Hodgkin; the new method consisted in drawing the outlines of the organs on a piece of lace, stretched on a frame and placed over the body, the sketch being transferred by laying the lace over a sheet of paper, with a piece of the "manifold letter-writer" paper interposed. By pressing firmly with a point on the chalked outlines they were traced in black on the paper beneath. This plan is applicable to the living as well as the dead subject. The full-sized diagrams were reduced to the size they present in the author's essay by the pentagraph.

In the first section of the paper, the "actual and relative position of the healthy organs" is examined; the lungs and heart being made the earliest subjects of consideration.

In describing the lungs "above the clavicle," the author observes that the pleura covering this portion of the organ "is strengthened and brought under muscular control by a fascia, the expanded aponeurosis of a small muscle, the pleural scalenus, arising from the transverse process of the last cervical vertebra, and inserted by a dome-like fascia into the whole of the upper edge of the first rib."

In the natural state, the inner edges of the lungs rapidly converge from opposite the clavicle, and usually come in contact at a certain point, which has long been known to vary with circumstances; but the author shows with precision what these circumstances are, and how they act. In the state of health and in the adult, contact takes place just behind the junction of the first and second sternal bones. In children, the thymus separates the lungs; and while the absorption of that body continues incomplete, the point of contact is lower than in the adult. In old age and in cases of emphysema, on the contrary, the place of union is higher than natural. After uniting in the manner described, the apposed edges continue for a short space in contact, separating again at a point ranging between the level of the third and fifth costal cartilages. In cases of pericardial effusion the bulging sac drives the lungs apart in an opposite direction, and raises the point of inferior separation.

In the inferior separation the right lung takes no direct part, as its inner edge passes vertically downwards behind the centre of the sternum, to the attachment of the ensiform cartilage. The inner margin of the left lung passes from the point of separation obliquely downwards to the left, usually taking the course of the fourth cartilage. In robust subjects this line is lower, and it is of course carried downwards by all pulmonary affections, (such as emphysema, bronchitis, pneumonia,) increasing the bulk of the lung. It is pushed slightly upwards by simple enlargement of the heart; considerably so when there is superadded pericardial effusion or adhesion.

The lower edge of the right lung is stated by the writer to pass usually across the conjoint cartilages of the sixth and seventh ribs, and then behind the fifth intercostal space; the lower edge of the left lung is commonly half a rib's breadth lower. The inferior posterior limits of both lungs lie just behind the junctions of the twelfth ribs to the vertebræ, and pass directly outwards and forwards. It is to be remembered that these outlines are of the organs as found in the dead subject.

In the author's minute description of the positions held by the various portions of the heart and the great vessels connected with it, there is little which can be considered as at once new and of practical utility. He observes, (and the fact is indubitable,) that the apex of the heart is almost always, after death, drawn upwards for about half a rib's breadth; we have known the correctness of the results of percussion questioned through ignorance of this point. Yet two pages after (p. 330) the author states that 'in life-time the lower bound [boundary] of the heart is usually about half a rib's breadth higher than it is after death." Here must be a typographical error.

Sounds due to mitral regurgitation are heard, though very faintly, to the left of the seventh and eighth dorsal vertebræ. Now as the aorta is interposed in this situation between the vertebræ and heart, aortic valvular sounds are also audible here. But the following particulars will suffice for their distinction, according to the author's statement. If the abnormal sound detected be also present and louder, opposite the third and fourth dorsal vertebræ, it is due to disease of the aortic valves; but if the sound become louder on approaching the heart's apex, and is not heard behind the upper dorsal vertebræ, it is due to mitral regurgitation. This point is likely to prove useful in cases where the *anterior* diagnosis (if we may so speak) of these affections is difficult.

Mr. Sibson finds that the arteria innominata lies "in front of the trachea, just above the sternum." The fact, that when above the sternum the vessel lies rather to the right of the windpipe, should be added, to render this description correct. Its pulsation is visible in cases of aortic regurgitation.

The author judiciously analyses the influence of the heart's action and of respiration on jugular pulsation. The mere existence of such pulsation, he concludes, is anything but an indication of disease either in the pulmonary valves or elsewhere. In diseases attended with impeded flow of blood through the lungs and heart, the jugular veins contain more blood and their pulsations "are more visible than in health;" but where the impediment is extreme, the veins are in a state of constant distension and no pulsation is visible.

Few points, if any, are possessed of greater interest in connexion with such inquiries as those we are examining, than the precise position of the right and left divisions of the diaphragm. The profession is indebted, as many of our readers are doubtless aware, to Dr. Edwin Harrison for an ingenious method (in the application of which he is himself, at least, eminently successful,) of determining the position of the divisions of the membranous septum in question; and also for the announcement (of which the experience of all careful observers has proved the accuracy,) that those divisions rise much higher into the thorax, than it was formerly the habit to believe. Mr. Sibson, using the more ordinary method of percussion for ascertaining the upper outline of the liver, finds that this usually rises as far as the fourth intercostal space, often even to the fourth rib; in children only to the fifth. Diseases enlarging the liver may carry the right side of the diaphragm as high as the third rib. The left side of the diaphragm in the natural state, as ascertained by Dr. Harrison, is at least as low, and commonly a little lower, than the right.

The position of the liver is materially influenced by the state of the stomach. If the latter organ be moderately distended, the former extends to the left about half way between the ensiform cartilage and the apex of the heart. It extends further in this direction in children and in women. On the other hand, when the stomach is empty, the liver falls over into the space filled by the former when in a state of repletion.

We pass over the author's very circumstantial analysis of the phenomena of inspiration and expiration, as neither remarkable for any useful novelty nor readily susceptible of condensation.

This writer exhibits himself, within certain limits, a partisan of the theory which assigns a laryngeal origin to the respiratory murmurs heard over the surface of the chest. In *tranquil* breathing the inspiratory murmur is, he conceives, doubtless due to the conduction of the laryngeal sound, and not to a new sound generated in the air-cells; else would the inspiratory murmur be loudest where the air-cells are most numerous, while in reality the reverse of this is the case. In tranquil inspiration the air, as it advances, occupies more space, moves slower and with less friction. In expiration, on the other hand, the air occupies, as it advances, a constantly narrowing space, moves quicker and with more friction,—conditions more favorable to the generation of sound; but, continues the author, the movement of the air is so slow, especially in adults, that no sound is produced.

In *hurried* breathing, on the contrary, Mr. Sibson thinks that the inspired air generates sound by friction against the sides of the tubes, small and large; during rapid expiration sound is excited in like manner, but as the air passes from a larger into a smaller space, it is often a shade louder than it is during rapid inspiration. These sounds, it is added, are (contrary to what is observable in the others,) as loud over the lower part of the lung as over the upper portion which is nearest the larynx.

The pith of this argument is readily seized, and it is not without ingenuity. But independently of the *primâ facie* difficulty of admitting that phenomena perfectly identical in nature, and differing only in amount and intensity, can be generated in altogether different ways, there is an argument urged by Dr. Walshe in his work on the Chest, (p. 188,) against the laryngeal theory, as originally taught by M. Beau, which appears to us (for we have ascertained the accuracy of the *fact* on which Dr. Walshe's objection rests,) likewise fatal to Mr. Sibson's modified version. In commenting upon *continuousness* of the inspiratory and expiratory murmurs of each respiration as an important character of healthy pulmonary respiration, Dr. Walshe observes: "This character would of itself be sufficient to announce the lung as the part ausculted; for it will be found that in proportion as auscultation is practised at a further point from the pulmonary parenchyma, so will the two sounds be more and more distinctly separated from each other by an appreciable interval of time, an interval which consequently attains its maximum opposite the larynx and upper part of the throat." Here the inference is clear: were the sounds, audible on the surface of the chest, merely the transmitted laryngeal murmurs, they should be separated from each other in each respiration by even a longer interval of time than that elapsing between the inspiratory and expiratory sounds opposite the larynx.

In noticing the long-known fact that the semi-circular measurement of the right side of the chest at the base of the lung exceeds, in adult persons, that on the left, the author ascribes the excess to the "liver being generally more bulky than the combined stomach and spleen." Now this explanation of the fact seems of questionable justness from the following circumstances: In children (as noticed by the author,) the two sides of the chest seldom differ; yet (as also remarked by Mr. Sibson,)

the liver is of disproportionately large size in them. Mr. Sibson has not taken into consideration the influence of excess of muscular exertion on the right side in expanding that segment of the chest;—an influence, the reality of which cannot be doubted, if the statement of Dr. Walshe, (Op. cit. p. 21,) that "in left-handed persons the left side sometimes measures more, or more frequently the same as the right," be correct. It seems to us probable that both the influences referred to exist, but that the latter is the more powerful of the two.

Mr. Sibson attacks the habit of tight-lacing on two grounds: first, as destructive of the beauty of female form; secondly, as productive of shortness of breath, palpitation, indigestion, hysteria, and of the excess of deaths from consumption between the ages of fifteen and thirty among females as compared with males. The queen, we are told, "rains healthy influence on the domestic manners of the day," and from this and the interference of Sir James Clark in the case of the Princesses, a general change, it is hoped, will ensue in regard of the use of stays. As respects the first point, we confess we hold no very decided opinion; however dressed, whether the figure be rendered protuberant by *tournure*, or balloon-like, with copious folds of *crinoline*, flattened or squared, lengthened or shortened, compressed or expanded (and upon some one or other of these modes of proceeding, the women of all countries seem agreed,) the effect is very much the same, the real outline of the shape is concealed. Well-constructed women suffer, the ill-shaped of their sex gain, by these practices; the latter are in the majority, the practices will consequently prevail. The particular one to be adopted seems to us a matter of little moment; besides, *de gustibus non est disputandum.* But in regard of the writer's statement concerning the influence of stays in generating consumption, we must express the opinion—or we should say the fact—that such influence is, in the existing state of knowledge, mere matter of hypothesis, plausible hypothesis we deny not, but still hypothesis. And we cannot help regretting that Mr. Sibson did not provide himself with some description of proof, before he gave additional currency to the fashionable figment. "*Il y a des choses que tout le monde dit, parcequ'elles ont été dites une fois,*" said Montesquieu, and the outcry against stays seems to us an excellent illustration of the apophthegm. However, we have done on this matter, having already (Brit. and For. Med. Rev. Vol. XVI, p. 454, Oct. 1843,) when noticing the incredulity of M. Louis in the same direction, given utterance to our convictions.*

*Emphysema.* In describing the anatomical characters of emphysema, the author incidentally attributes the opinion that the disease is a "hypertrophous" one to Andral; this is an error, Andral (Anat. Pathologique, t. ii, p. 526,) in the most distinct manner refers the affection to atrophy of the tissue of the lung.

The slowness and difficulty of expiration in emphysema are well

* Mr. Sibson, like those who have before him taught his thesis *more medico*, does no more than give a prosaic and less convincing version of the poetic expostulations of Mirabeau with his mistress (Lettres à Sophie; Lettre seizième,) on the evils of whalebone encasement. But the passionate lover was more reasonable than the *sang-froid* measurer of chests; he nowhere ascribes the generation of consumption to the use of stays, but limits himself to an energetic denunciation of their influence in producing hysteria, dyspnea, and similar states, much more demonstrably their real effects.

known; Mr. Sibson, with considerable plausibility ascribes the peculiarities to the fact of the communications of the dilated cells with the bronchial tubes being very small in comparison with the size of those cells.

Prominence of the post-clavicular region has long been noted among the signs of emphysema. The author observes upon this point, that the summits of the lungs are raised, but not so much, proportionally, as the clavicles; the apex of the lung does not consequently rise so high above the clavicle, as it does in the healthy state.

Mr. Sibson is more precise in his account of the depression undergone by the diaphragm in this disease than his predecessors. It is carried so far downwards that the lower margins of the lungs lie in front behind the sixth intercostal space, or the seventh rib. The lower inner front margin of the right lung, and the lower border of the heart fall to behind, or even lower than the ensiform cartilage. The lungs at the back reach to just below the lower edge of the twelfth ribs. The deepest inspiration of health does not carry the lungs to these extreme limits, nor can they ever almost be reached by the utmost degree of artificial distension in the dead body.

Even in the most advanced forms of the affection, there always continues to be a space discoverable in the cardiac region, furnishing a dull sound; the distension of the right cavities of the organ, which appears constantly to attend the pulmonary malady when strongly marked, probably accounts for the persistency of the dull sound.

The heart being lowered, the vessels connected with it undergo depression also; for instance, the "innominata is lowered, and (the sternum being raised,) is entirely within the chest, behind the sternum, and quite out of the surgeon's reach." It may not then be a matter of indifference to the operating surgeon to understand the diagnosis of emphysema.

With respect to semicircular measurement, the right and left sides are said by Mr. Sibson to be "almost always equal." It would be more correct to say, as M. Woillez did many years since, that the average excess on the right side is much less in emphysematous than in healthy persons; at least this is what we have ourselves also found to be the truth. The fact proves by inference the greater frequency and amount of emphysema on the left than the right side.

Emphysematous patients, if in bed when attacked by a fit of dyspnea, frequently place themselves on their knees and elbows, leaning the head forwards on the pillow. In this position, remarks Mr. Sibson, "there is no occasion to lift up the thoracic walls on inspiration; the motion is backwards and forwards, like that of a battering ram, and the costal walls are slung by the serrati from the scapulæ, fixed by the rhomboidei. The action of the serrati, when exerted on the ribs, is not inspiratory, but expiratory; so that expiratory action is also assisted. The head is at the same time lowered; the origin of the scaleni and of the sterno-mastoidei are thrown forwards and fixed, and these muscles act with every advantage; their pull being direct, not oblique, none of their force is lost." This is a neat physiological analysis of a well-known symptomatic state.

Diminished intensity of transmission of the heart's sounds through the pulmonary tissue affected with emphysema, had been noticed by Dr.

Walshe as a sign of some value in that disease. Mr. Sibson's inquiries, confirmatory of those of the author just named, are original in having been directed to the distinction of the effects of inspiration and expiration on the cardiac sounds. On a deep *inspiration*, he says, the sounds are lowered, with the region of the heart's dulness and impulse, into the epigastrium. The systolic "impulsive" sound is louder and more ringing than in the tranquil state, probably in consequence of the falling in of the sterno-costal walls over the heart, while that organ advances. The diastolic sound Mr. Sibson has discovered to be generally louder, sharper, and more ringing than in the natural state. Both sounds are only audible over and in the immediate contiguity of the seat of the heart's dulness. On a deep *expiration* the "impulsive" sounds are loud and ringing, and heard more extensively than during inspiration. It will be observed, however, that these variations in the intensity of the cardiac sounds, dependent on inspiration and expiration, cannot be considered in anywise characteristic of emphysema; differences, the same in essential character, exist in health.

Mr. Sibson has found the frequency of respiration generally natural, or even below the healthy standard if there be no bronchitis, and the patient be in a state of repose. According to his experience the pulse is almost always slow in males, even when the respiration (from attending bronchitis) is quick; it varies from forty to seventy in a minute. In the female the pulse is quicker, varying from eighty to ninety: the breath also is, relatively to the pulse, quicker than in males. These differences in the female sex are ascribed to the influence of tight-lacing.

Mr. Sibson expatiates on the causes of emphysema. At the head of the list he places bronchitis; Laennec had done so before him. M. Laennec's doctrine has been shown by Louis and others to be irreconcileable with facts, and Mr. Sibson's pages contain no novelty in its support. Next follow diseases of the heart, frequent exposure to foul air, repeated excessive exertion, such as playing on wind-instruments, &c.; the grounds for ascribing the affection to these agencies are merely theoretical, and hence undeserving of notice; why did the idea not occur to Mr. Sibson, that it might be well to show that wind-instrument players were actually more subject than other persons to the disease. For our parts we doubt the fact, and who can gainsay us? Again: "affections of the stomach and liver, caused as they *generally* are by a deranged condition of the skin, *frequently* give rise to repeated excessive attempts at a deep inspiration and induce emphysema!" Fortunately for Mr. Sibson's reputation, but little matter of this purely fanciful and really empty character is found to sully his essay.

*Bronchitis.* Mr. Sibson is likewise a micrographer. He details the microscopic changes in bronchitis, to the consideration of which disease we now bring our readers. "The larger bronchial tubes were covered on their inner walls with irregular elevations, composed entirely of capillary vessels. The vessels are large and very irregular in caliber, the same vessel becoming suddenly two or three times its previous size, and then shrinking suddenly to a bore even less than it had at the first start. Some vessels were twisted spirally; others formed loops, rising forward; and between others irregular reticulations were formed by capillaries of ever-varying, though small, size. Covering one portion of this minutely injected tissue, there was an opalescent, soft, feebly-organized structure, floating here and there in filamentous ruggedness; under the microscope this structure was seen to consist of parallel bundles of exceedingly fine lines, each line being many times finer than a blood-globule. The enlarged looped blood-vessels played in a thick, soft, smooth, nearly translucent membrane. The vessels did not generally penetrate quite to the surface of this membrane; here and there one perforated it, and was truncated."

Diseases where "one lung and one side of the chest are amplified," next engage Mr. Sibson's attention; these diseases are spoken of as pneumonia, pleuritis, and diffused tubercular consolidation. In diffused enlargement from any one of them, the organ, or portion of it, affected, "puts on the same form that it acquires on a deep inspiration."

The explanation of dilatation in such diseases has been usually mechanical—from the first observations of Broussais on the subject downwards. Mr. Sibson, on the contrary, holds that "when any one of the diseases in question occur, especially in an acutely inflamed stage, the muscles of inspiration act to expand the chest over the diseased part, and to *withdraw from that part injurious pressure.* The dilatation from inspiration so effected is almost or altogether permanent, to stave off from the inflamed parts the injury that perpetual friction would cause." We confess we have no little difficulty in conceiving how a limited portion of the walls of the chest can maintain itself persistently distended, while the subjacent part of the lung still undergoes any degree of respiratory change of bulk; the thing indeed seems physically impossible.

*Pneumonia.* The anatomy of pneumonia is minutely described, and the following general statement of the anatomical stages of the disease deduced.

"First stage. The cell-walls are unusually minutely injected; the large filamentous and the small webbed interlacing capillaries are irregularly large and tortuous. Second. The interlacing capillaries are either irregularly large and tortuous, and end abruptly after a bulge, being clogged by secretion; or else they are unusually small and gradually fine off to a point. Third. There are no capillaries whatever injected, or very few; the texture of the air-cells is thickened. The injected walls of the pulmonary artery, and of the large bronchial tubes, run for a short distance into the diseased structure, and then end abruptly. Fourth. There are no injected capillaries, and granules occupy the cells. Fifth. When there is hepatization, the pulmonary arteries are obliterated, forming white tortuous-looking, ramifying cords. The pulmonary veins are collapsed. The cells are distended with hard, reddish-brown, friable structure. The cells themselves have not now the smooth defined character, under high microscopic powers, that they had; their walls are shaggy; the very fine filamentous texture, seen in the previous stage, becomes less definite, and is, as it were, apparently in strings of infinitely small beads or cells. Sixth. The stage of splenization, of breaking up of the cell-structure, of complete softening and disorganization of the diffused granular structure, and of the gradual disappearance of the blocked up capillaries. The obliterated pulmonary arteries collapse and remain in the form of tendinous filaments."

There is much that is correct and valuable in this description; but there is an important omission, not a syllable is said of the condition of the inter-cell structure of the organ, the presence of exudation within its meshes, &c., so that actually this structure would appear in the mind of the author to be (unless as regards the vessels permeating it, and the changes going forward in the adjacent cells,) unconnected with and uninterested in the phenomena of pneumonia.

The next page startles us with the announcement that Mr. Sibson has "observed" pneumonia to end in universal tuberculous infiltration, strengthened as the averment is by the statement that this is "by no means an unusual result." We do not contest Mr. Sibson's having in some rare instances observed the termination in question; accurate physicians on the continent and here have done so also, but we should delight to be informed how or when he has ascertained that such termination is a common one. Meanwhile we refer him to M. Louis's work on 'Phthisis,' (2ème edition, p. 595. Paris, 1843,) for a series of facts collected from his own observations, and that of the patient and laborious M. Grisolle, demonstrating the excessive rarity of the connexion now spoken of between pneumonia and phthisis.

Cases of pneumonia are occasionally observed, as has long been known, in which there is complete absence of respiratory murmurs, rhonchus, and vocal resonance—a state assimilating such cases, in respect of physical signs, to extensive pleuritic effusion. The fact is indubitable; the difficulty consists in explaining it on the admitted doctrines of transmission of sound through solid lung from the larger bronchi. Mr. Sibson simply says, (like those who have preceded him,) "the bronchi are no longer open tubes; the respiratory sounds and the vocal resonance are no longer conducted by them to the surface." This is obviously unsatisfactory. Skoda makes much of such cases. It is matter of belief, and we think not incorrectly so, that suppuration leading to elimination of pus through the bronchi (qy. hepatization, with breaking up of structure) is announced by a variety of humid rhonchus, sub-crepitant, mucous, gurgling, as the case may be. Mr. Sibson goes further than this: "if an empty cavity is formed there is cavernous respiration, and the modification of vocal resonance produced by the accompanying hissing, cavernous respiration." We cannot believe this *possible* in connexion with pneumonia, (Mr. Sibson has obviously never *observed* it;) and such diagnosis of pneumonic abscess seems to us likely to remain a matter of paper, and not clinical, acquirement.

There is nothing else new in Mr. Sibson's account of this auscultatory phenomena of pneumonia: he sides with those who consider the respiratory murmurs altered in character before crepitation becomes audible, and places the seat of production of the crepitant rhonchus in the interior of the air-cells.

*Pleurisy.* Mr. Sibson, describing the physical condition existing in pleuritis, remarks that the "walls of the chest are drawn away from the inflamed lung-surface; that of course that surface follows; but that the *pressure* exerted by the walls of the chest is *diminished.*" We must beg to differ from the author here; the proposition just quoted makes it matter of inference that in the healthy state reciprocal pressure of some amount exists between the lungs and thoracic parietes—a notion as inadmissible through observation as it is opposed to the physical principles regulating the mutual play of the lungs and their containing case.

That pleuritis is more frequent as an accompaniment to other diseases than as a simple affection, is a fact, in the announcement of which Mr. Sibson is less original than in the statement that the character of the secondary pleuritis "closely resembles, and is usually, in general principles, identical with that of the primary *exciting* affection." In respect of phthisical pleurisy, the position in question has, it is said, been long considered established; the points upon which the similarity is avowed to rest in regard of other diseases are microscopical, to our minds not very satisfactory, and at all events must be sought for in the essay itself.

Mr. Sibson pronounces pleuritis to be a very frequent consequence of emphysema. It has been on the contrary numerically proved by M. Louis (Mémoires de la Société Médicale d'Observation, t. i,) that pleuritis is not more frequent in persons dying emphysematous than in those free from this affection; and further, that when such pleuritis is present it cannot legitimately be considered an effect of the diseased state of the vesicles.

"The affected side is not so much larger than the sound side in pleuritis as in pneumonia:" here is a statement which might puzzle the author's readers very considerably. It is meant, of course, to refer to cases of pleurisy in which effusion has not yet supervened.

The following contrast is drawn between the distension produced by pneumonia and by pleuritic effusion. We transcribe the passage, though we must affirm the author's tendency is rather to exaggerate the amount of dilatation actually witnessed in practice as a consequence of parenchymatous inflammation.

"In distension of the chest from pleuritic effusion, the intercostal spaces are on a level with the ribs. In pneumonia, the distension of the chest and the displacement of the viscera are identical with the distension and displacement from a deep inspiration. In pleuritic effusion of fluid or air the distension is in every direction; the bulging does not take on the form of the lungs, but it presents a general somewhat globular distension. The superior ribs are separated from each other; in pneumonia they approach. [?] In pleuritic effusion, the mediastinum and heart are thrust over to the opposite side; in pneumonia, the displacement of the mediastinum and heart is comparatively slight. In pleuritic effusion of the right side, the lower edge of the liver is pushed down considerably below the eleventh rib; the whole liver, in extreme cases, is thrust down from behind the cartilages of the ribs. In pneumonia, the lower portions of the liver alone protrude beyond the cartilages, the bulk of the organ being still behind them."

Percussion over a pleuritic lung is, before effusion occurs, usually quite as resonant as on the healthy side; this is the opinion held by writers in general. But Mr. Sibson has "*observed* it to be now *more resonant.*" We should have wished to be informed how often he has succeeded in ascertaining this, and above all, how he satisfied himself that the state of the other lung did not lead him into error. For our own parts, we have much less difficulty in believing (granting this fact to have been actually observed), that greater comparative clearness of sound over an inflamed pleura arose from the existence of pre-existing slight consolidation on the *other* side, than that it arose "from increased volume of the lung on the pleuritic side, owing to the universal expansion of that side, compensating [but it does more than *compensate*, according to the author,] for the dulness that the comparatively slight consolidation over that lung would occasion." It is to be noted that here is involved not a mere nicity of diagnosis, but to a certain extent a matter of practical import—the distinction of pneumonia and pleurisy at a very early stage.

During the stage of plastic exudation (before the occurrence of liquid effusion), the pectoral resonance is, if anything, "louder over the pleuritic lung;" we should infinitely prefer to find Mr. Sibson adding the number of times he has actually observed this, rather than giving the statement a quasi authenticity and force by the argument, "*as* that lung is the larger, the volume of the vibrating mass of lung is increased." On our own side, though we have certainly never observed the alleged increase, we have no desire to question its possible occurrence, for we cannot affirm we have specially examined the point.

Mr. Sibson ventures on a new explanation of the variety of vocal resonance, known as œgophony. "The pectoral resonance," he says, "is accompanied by the whispering friction-sound; the two sounds are heard together, just as the drone and the notes of the bagpipe are; the person seems to be whispering and speaking into the ear at the same time; a peculiar tremulous or bleating vibration is produced, in fact, œgophony." Objections might be made to this mode of explaining the peculiar character of œgophonic resonance, but we nevertheless consider it worthy of serious consideration.

It has never fallen to Mr. Sibson's lot to hear friction-sound satisfactorily in emphysema; he considers it disputable whether the hissing noise over the greatly-dilated marginal cells be due to friction-sound or to the entrance of air through narrow openings into large sacs, and inclines to refer it to the former. The opinion that friction-sound might be generated in emphysema, independently of pleuritic exudation, was originally put forth by Laennec, rejected by the majority of subsequent observers, and revived, if we mistake not, in a modified form by Dr. Walshe. It appears to us, with Mr. Sibson, that the point is one still debateable; but as we have more than once referred to it in these pages, we shall at present not delay longer in its consideration.

Phthisis "never" goes through its stages without abundant pleuritis, according to Mr. Sibson. The words "never" and "always" should be banished from the vocabulary of pathology. The truth is, in regard of this point, that phthisis does run to the last without the formation of adhesions (and without these the diagnosis cannot be regarded as scientifically certain), in one of every hundred cases, or thereabouts, as proved by M. Louis (Op. cit. p. 42.) The writer adds, that very often the lung that is least diseased gives evidence of pleuritis by a modified friction-sound over the upper part of the chest, which sound is often styled puerile respiration, and closely resembles this. We believe, with Mr. Sibson that friction-sounds are oftener heard at the apices of tuberculous lungs than either the admitted theory of the generation of such sounds, or the statements of authors would lead us to suppose; but we question the fact of such sounds being confounded with peurile respiration.

*Phthisis.* A new section introduces us to diseases in which the bulk of the affected lung is lessened. At the head of the list (or, indeed, rather constituting here the only illustration of such lessened bulk) figures phthisis. Now it will be remembered that tuberculous infiltration was one of the conditions set down by Mr. Sibson as productive of enlargement of the lung—a statement we purposely forbore commenting on at the time, much as we were surprised at its appearance. It would now appear that Mr. Sibson has only *imagined* the enlargement in question; for he states in the present place "when phthisis is so far advanced as to be recognized, the affected part of the lung is lessened." No doubt it is, and, as clearly as logic is logic, Mr. Sibson forfeits his character for soundness by setting down fancies concerning what exists at a period when neither he nor any one else can prove the nature of the complex phenomena going forward. We have an obscure memory of having read somewhere else this opinion, that at first the lung undergoes increase of bulk in phthisis, but where or by whom enunciated we cannot call to mind. It is to be observed that cases where the entire lung is consolidated, and where inflammation exists, and actual inflammatory products are present, are not justly to be included in the category of common phthisis; of enlargement under such conditions Mr. Sibson has figured an example.

Mr. Sibson places the seat of development of tuberculous matter in every one of the tissues of the lung. This is in consonance certainly with reason, and, we are satisfied, with observation. He adds another to the multitude of microscopical accounts of tubercle which have of late years besieged pathology,—it appears well considered, and to be founded on careful observation; but we should be glad to know how the author ascertained, satisfactorily, the hollow or cell character of "almost point-like" bodies scattered in numbers over the surface of certain cells of the $\frac{1}{1000}$th of an inch in diameter, and even of others seated upon the largest of these "secondary cells." An enumeration of the points of analogy and difference between tuberculous disease and pneumonia follows the description of the microscopical characters of the former; from this comparison the following is the general inference: "There is then a range of analogies, but a vital difference between the two diseases; they both infest the same structures; they both work out their mission by parallel steps; but the steps only resemble, they are not alike. They are alike in this: each requires for its development the assistance of capillaries; in each new capillaries are formed, when there are no old vessels to give the required aid; in each, an inflammatory action is necessarily involved, especially in the pulmonary artery, the bronchial tubes, and the pleura.

In phthisis the least diseased lung is the larger of the two: Mr. Sibson appears to think (as already mentioned) that it is only generally so, and not in all instances. His mode of accounting for the excess of size on the comparatively sound side is sufficiently curious: "Active pleuritis, without adhesions, exists in the early stage; the walls of the pulmonary artery and the bronchial tubes are inflamed, and the air-cells are infiltrated in patches, probably inflamed.......... The pneumonia and pleuritic parallel conditions lead to an undue permanent expansion of the lung.........I think it reasonable to infer that the walls of the chest are more expanded over the seat of tuberculous disease and tuberculous pleuritis, in the very early stage than they are over the healthy lung." This is an excellent example of hypothetical reasoning, founded on a postulate (the existence of pleuritis) against the admission of which common experience protests.

Mr. Sibson affirms that there are "many" cases of phthisis, where the tuberculous matter does not extend to the surface of the lung, and where cavities have not formed, which can only be detected by the diminished play and bulk of the affected organ—cases in which percussion, auscultation, and the general symptoms convey no information.

We are not disposed to make light of the diagnostic value of defective motion and depression of the walls of the thorax in phthisis; but we confess we have yet to meet with a case in which these were sufficiently marked to justify the observer in pronouncing tubercle to be present—the other modes of investigation referred to failing to throw any light on the nature of the disease.

An inquiry is next made as to the cause of the bulk of the affected portion of tuberculous lung diminishing before the stage of softening and evacuation of the morbid structure. The diminution is referred to the non-expansion of the unaffected air-cells; and we are further told that, "wherever inspiration would injure an affected lung or portion of a lung, or cause suffering to the adjoining diaphragm, costal walls, or parts contiguous to those walls, then breathing is locally suspended, and that lung is in a state of repose." That the above explanation is incorrect as a matter of fact, follows obviously from the observations of several pathological anatomists, who have shown that a special form of emphysematous distension (instead of "non-expansion,") is extremely common in the cells adjoining tuberculous deposit, and that other conditions (to which we can do no more than allude here,) are the real agents in effecting the diminution of bulk. Secondly, as to the suspension of breathing, which is presumed to lead to the permanent non-expansion in the manner stated, we appeal to any one in the habit of seeing thoracic disease, whether they believe in its reality.

Mr. Sibson maintains that the volume of the chest does not necessarily lessen *step by step* in the formation of cavities; this we know to be correct. But we have never observed a case justifying the statement that, "on the contrary, the advance of the disease, the formation of new tuberculous and puriform matter, often *adds to the bulk.*" That such is not the usual opinion, we need scarcely inform the reader; and it appears as well to let him know that Mr. Sibson adduces no decided evidence of the accuracy of the new doctrine.

When a cavity is formed, where a solid block of tubercle or an accumulation of puriform fluid previously had place, the volume of the lung lessens, and the respiratory play of the superjacent walls is increased.

The author has found the heart commonly, but by no means invariably, lessened in bulk when the lungs have undergone a similar change. Nevertheless, an unusual portion of the cardiac surface comes in contact with the walls of the chest. There is nothing new in the statement, that the heart's impulse may sometimes be felt so high as the second intercostal space.

We must pronounce Mr. Sibson's attempt to describe the particular characters of phthisical, as distinguished from other kinds of dyspnea, to be a failure; but, in extenuation of his want of success, we feel it just to add, that accurate verbal description seems to us an impossibility. It is certainly not true, as a general proposition, that the shortness of inspiration in consumptive persons depends on the expansion of the chest "being suddenly arrested by a stitch in the side—a sudden muscular pain:" this is the exception, not the rule.

In the early stages of phthisis, the author detects a comparative dulness of sound under percussion, over the affected part of the lung. This dulness, he does not doubt, depends on turgescence of the capillaries preceding the earliest grayish infiltration; after a time (that is, when tubercles are actually formed, but scattered and not numerous), it disappears: hence "the lung, in the incipient stages, will be less resonant than in the more advanced stages." *Credat Judæus,* might, on first sight, appear to be the only fitting comment on this announcement. It is not, however, absolutely devoid of some claim to attention. In the first place, it is no doubt conceivable that intense congestion of a portion of lung shall affect the resonance under percussion to a greater degree than a few scattered tubercles, especially as we know the most skilful persons admit the impossibility of detecting any change in the resonance, as a constant result of the latter condition. But such congestion is not the anatomical deviation from health Mr. Sibson has in view; and even if it were, the task would remain for him of proving its occurrence—not its uniform occurrence, (for he could not demonstrate what does not exist,) but even that it precedes the formation of tubercle in a moderately large fraction of cases. The idea of his "capillary turgescence" producing an appreciable degree of dulness, seems to us in the last degree problematical. Again, observe the contradictions in which the author involves himself: he affirms, as we have seen in a previous page, that accumulation of tuberculous matter may exist to such extent as to cause diminution of the bulk of the organ, with impeded motion of the thoracic walls, and yet not affect the clearness of the sound under percussion; in the present passage he maintains that turgescence of the capillaries of a limited extent of substance produces dulness of sound. Is it in reason to be conceded, that such capillary injection and turgescence can be detected by fingers and ears which fail to discover an amount of tuberculous formation producing the changes in the lungs and parietes referred to? Lastly, as the truest test of the point, we would ask Mr. Sibson how often he has observed the phenomenon he describes, and how he assured himself in each instance that no accidental circumstance may have been present to modify the physical signs.

"Vocal resonance," says Mr. Sibson, "is in general unusually feeble over the lessened, partially condensed, tuberculous lung, prior to the formation of a cavity." Here is a flat contradiction of accepted doctrine.

The causes and general history of the first symptoms in phthisis all tend to prove, in Mr. Sibson's mind, that it is essentially a disease involving inflammatory action. No new proof of this notion (which has been refuted even in this Journal, *usque ad nauseam* almost) is adduced by the author; the statement does not consequently call for particular examination.

Mr. Sibson suggests that the sudden evacuations of blood from the lungs which are so common in the later stages of consumption, "are doubtless due to softening of the walls of tuberculous cavities, which are vividly supplied by blood-vessels." He "doubts not that the walls of the pulmonary artery often give way by the onward spread of the degenerative structure." Of these two modes of accounting for the symptom in question, the first is open to objection on the ground of the well-known fact that copious hemoptysis is far from unfrequent in the earliest period of the disease, and is comparatively rare at the close of existence. (See Louis, Op. cit. p. 197.) The other is utterly unsupported by observation; it is matter of notoriety, as admitted by Mr. Sibson himself, that the detection of an open vessel is singularly rare.

As an illustration of the author's remarks upon "other diseases in which the lungs are wholly or partially diminished," we may condense his account of "diminution of the bulk of the lungs, narrowing of the chest, and elevation of the diaphragm from frequent irritable cough :" the statements made are at least worthy of being submitted to the clinical test. Many persons whose lungs are far from disease are subject to distressing cough, says Mr. Sibson, from the slightest exposure to cold, or from the influence of a full meal or an empty state of the stomach. An attempt to take a full breath even will bring on an attack of coughing. The result is a gradual diminution of the bulk of the lungs; the chest becomes narrowed, the diaphragm is usually raised, and the abdominal organs become disproportionately large.

The heart in the state of health forms the next subject examined in the essay. By inserting a graduated cone into the vessels or outlets of the heart, the author ascertained their respective diameters. He also injected the cavities of the organ with plaster of Paris, cutting out the casts formed by the plaster when hardened, dipping them into water and ascertaining how much water they severally displaced. He did not arrive at a perfectly accurate estimate of the relative capacities; this was to be expected,—experiments of the same kind made by Portal, Legallois, and Meckel, with water and mercury, he might have known utterly failed in leading to a definite result. He considers the heart of a girl, aged fourteen, to furnish a fair general criterion of the comparative capacities in question; and in this instance the casts of the right and left ventricles displaced the same quantity of water. In Meckel's experiments, on the contrary, the right ventricle was always found to contain much more mercury than the left, varying between *three times* the amount and an eighth of the amount.

Mr. Sibson has observed the motion of the exposed heart of asses poisoned with wourali,—artificial respiration being kept up in the ordinary way. The account given is interesting, carefully set down, and worthy of perusal in the original; we cannot afford it space.

In enumerating the effects of arresting the flow of blood through the pulmonary artery in these animals, the author states that as soon as eight or nine contractions of the heart had taken place after pressure had been applied to the vessel, the right auricle appeared to be quite empty, the ventricle swollen but not distended, and "regurgitation was evidently permitted." The latter point is of obvious interest in its application to cases of disease in which pressure may bear specially on the pulmonary artery, while the tricuspid valve continues, in respect of its *anatomical* state, capable of performing correctly its ordinary functions.

The following account of the change of seat of the impulse of the heart induced by change of posture of the individual agrees so closely with what we have ourselves observed, that although it is founded on the examination of but one adult male, we transcribe it. When this "man lay on his back, the impulse of the apex was felt between the fourth and fifth ribs, just below the nipple; the fifth rib was slightly heaved up. When he lay on the left side, the apex was felt beating strongly between the fifth and sixth ribs, an inch or more to the left of the nipple. When on the right side, the impulse of the apex could not be felt; there was gentle heaving of the lower part of the sternum. When he lay on his abdomen, the apex was felt to beat over the third and fourth intercostal spaces. When he sat up, the apex descended from the fourth to the fifth intercostal space."

In distension of the pericardium with serous fluid, the sac acquires a pyramidal form, the apex corresponding to the upper part of the sternum. The high elevation of the dulness produced by this cause distinguishes it from that significant of hypertrophy of the heart, which, be it ever so marked, does not give rise to dull sound on percussion in so elevated a situation as that mentioned. These facts, long known, have been well illustrated by Mr. Sibson's experimental injection of fluid into the pericardial sac. The loss of the peaked form in the dull sounding part is one of the earliest signs of diminution of the effused liquid; with the further advance of absorption, the right boundary draws nearer and nearer to the sternum; the left border approaches towards the middle line, and the lowest dull sounding part becomes resonant from the stomach being allowed to resume its natural position. Such is the series of changes indicative of the most favorable termination of the disease attended with pericardial effusion. But if the upper border, after it has gradually descended with a peaked form, become stationary above the natural upper limit of cardiac dulness and lose the peaked form; if the right border become stationary, while the left passes further and further outwards, the lower border not regaining completely its natural elevation, the supervention of hypertrophy may be considered the cause of the deviation from the previously described series of changes. Again, if the upper boundary, after descending for a time, become stationary, lose the peaked form, and then again ascend; if the extent of dulness increase towards the left side of the chest, while the right boundary of the dull sound, after having returned inwards, retrogrades, and the lower boundary do not resume its natural position, then we are justified in concluding that enlargement of the heart with pericardial adhesions has taken place. Mr. Sibson exhibits these various facts to the eye in some diagrams of extreme interest.

That accumulation of fluid in the pericardium (especially when the result of inflammatory action) leads to visible bulging of the pericardial region, is a fact with which M. Louis long since made pathologists acquainted. But that the local prominence, thus induced, bears particularly on the second costal cartilage, is a statement of Mr. Sibson's, and, to us at least, new. This writer appears to regard the bulging in cases of pericarditis as the effect of accumulation of fluid only; but it has of late been contended, with no small plausibility, that the alteration of shape sets in even before fluid has been thrown out.

Mr. Sibson points out certain peculiarities of the precordial prominence, which seem to distinguish it as the result of hypertrophy, with pericardial adhesions, rather than of simple hypertrophy alone. "If the heart be enlarged, with adhesions," he says, "the lower two thirds of the sternum continue to protrude; the separation of the costal cartilages and ribs from each other rather increases; the sixth, seventh and eighth ribs project even to the left side; the whole of the lower left ribs are raised, and thrown outwards; the whole of the left side projects; and the sixth, seventh, and eighth conjoint cartilages to the side of the xyphoid cartilages, are much raised." On the other hand, in cases of hypertrophy alone, simple bulging over the third, fourth, fifth, sixth, and seventh costal cartilages is the only modification of external form to be detected.

In the early stages of pericarditis, when the ordinary friction occurring in the sac is not sufficient to produce any variety of rubbing sound, Mr. Sibson has found such sound may be caused by pressing gently on the costal cartilages or sternum with the head of the stethoscope; this pressure, he presumes (and the presumption does not appear to us free from difficulty,) displaces "any fluid that may be interposed between the costal walls and the surface of the heart," and so ensures close contact of the apposed surfaces. In order to apply this pressure with less uneasiness to the patient, he inserts a thin plate of wood (the head of a chip pill-box,) into the mouth of the bell of the stethoscope. Here is a hint which may perhaps be made useful in practice.

When considerable effusion of serum takes place into the pericardium, the heart is raised and its bulk lessened; the friction-sounds are heard a little higher in the chest under these circumstances, and are not audible over the lower part of the sternum or below the fifth rib. With the diminution of the fluid, the site in which the sounds are audible becomes lowered. Inspiration usually draws down the heart, and lowers and renders narrower the seat of friction-sounds; sometimes it prevents them altogether from being heard.

*Hypertrophy without pericardial adhesions.* The descent of the heart which is known to take place in this disease, should always be considered in connexion with the altered position of the various cardiac orifices, and of the great vessels which, as a matter of necessity, it entails. The precise bearings of the valves to each other cannot be stated; it may be said that they usually retain their relative positions to each other, but this varies with the amount of general displacement downwards. If the left ventricle be greatly enlarged, the mitral valve is pushed considerably to the left.

It is a mistake to suppose that the extent of cardiac dulness affords a precise measure of the amount of enlargement of the heart, and this because the lungs, which are often emphysematous, overlap the organ to an unusual extent. Forcible percussion even will not always protect the observer from this source of error. The extent to which the lower border of the cardiac dull sound is brought downwards, furnishes a better test of the amount of hypertrophy, especially in males; but even this is fallacious in certain cases.

If the right ventricle be enlarged and its walls thickened, the lower half of the sternum, the ensiform cartilage, and the left costal cartilages, from the third or fourth to the seventh, are, according to Mr. Sibson, heaved gently and steadily forwards, "not by a pointed impulse, but by a diffused steady advance;" the "protruded walls usually fall back quickly towards the end of the systole." Such a state of things as this, we can have no hesitation in affirming, must, to say the least, be singularly rare.

*Pericardial adhesions.* Mr. Sibson has seen four loosely-adherent hearts, of natural size, free from valvular disease; the individuals to whom they belonged having evinced no signs of cardiac disease, and succumbed to affections totally unconnected with the heart.

If the adhesions be dense, strong, and contracted, and unaccompanied by valvular disease, they may lessen the bulk of the heart's cavities, and impede their expansion. The shorter the adhesions, the more prejudicial must they prove. The costo-sternal walls, are dragged back as the ventricles contract, by some of that force which should be expended on circulating the blood; the cavities are not perfectly emptied, the columnæ carneæ prevented from contracting efficiently, and hence regurgitation will ensue in some instances, where actual disease of the valves does not exist.

The extent of the heart's superficial dulness is very great; its margin rises as high as the third costal cartilage, descends below the middle of the ensiform cartilage, extends about a rib's breadth beyond the right border of the sternum, while its left boundary is seated almost close upon the outer wall of the chest. Deep inspiration has little of the lowering effect on the position of the heart's dulness, which it is observed to exercise in cases of simple hypertrophy.

The following description of the impulse of the heart in such cases, accords, with trivial exceptions, with what we noticed in an individual recently submitted to our observation: "The impulse is felt over the entire region of pericardial dulness; that of the apex is extensive, strong, and thrusting, and is perceived very far over to the left side; in one case, the apex rose at the beginning, and fell back during the continuance of the systole. The sternum, costal cartilage, and xyphoid cartilage are heaved forward firmly and steadily at the beginning of the systole; and during its continuance those parts fall back steadily and quickly, coinciding with the mode of systolic contraction of the right ventricle. In some cases, the sternum and costal cartilages spring forward with a jerk during the diastole." In some instances, the fifth and sixth ribs and costal cartilages are drawn to the left during each systole, being dragged, as the author naturally supposes, after the heart, during its movements from right to left, by the adhesions.

When the pericardial adhesions are long and loose, and the heart of natural size and free from valvular disease, no physical sign announces their presence; and we have already seen that, in respect of local function or of the general health of the individual, they are perfectly innocuous.

Mr. Sibson "refers to his diagrams with satisfaction and confidence;" and he is fully justified in doing so. They supply the most convincing proof of his industry, and give a value to his descriptions which can only be fully appreciated by those who study his paper. The amount of information contained in the essay is not to be estimated by the notice we have now concluded: the construction and nature of the paper rendered general condensation of its contents a matter of impossibility, and we have consequently been obliged to pitch upon, rather than select, certain passages for comment, calculated to give a general notion of the nature and scope of Mr. Sibson's researches. If we shall have induced any of our readers to undertake the careful study of these in the original, the motive we had in view will have been fully secured.

It cannot fail to be observed, even from the analysis we have given, that the title of Mr. Sibson's paper conveys a very imperfect idea of its contents. Were we to be as critical in regard to style, as we have been in regard to matter, we could also point out some literary deficiencies; these are, however, but the dross, which cannot conceal the sterling gold from those willing to look for it.

